# Open Retroperitoneal Inferior Vena Cava Cannulation for Distal Ventriculoatrial Shunt Catheter Placement

**DOI:** 10.7759/cureus.21555

**Published:** 2022-01-24

**Authors:** Christian Mustroph, Sepehr Saberian, Katelyn Burch, Paul Parker, David Wrubel, Michael Sawvel

**Affiliations:** 1 Neurological Surgery, Emory University, Atlanta, USA; 2 Neurological Surgery, Morehouse School of Medicine, Atlanta, USA; 3 Pediatric Neurosurgery, Children’s Healthcare of Atlanta, Atlanta, USA; 4 Pediatric Surgery, Children's Healthcare of Atlanta, Atlanta, USA; 5 Pediatric Neurosurgery, Children's Healthcare of Atlanta, Atlanta, USA

**Keywords:** csf diversion procedures, csf diversion, ventriculoatrial shunt, ventriculoperitoneal shunt, hydrocephalus

## Abstract

Multiple alternative sites for distal ventriculoperitoneal shunts have been described including pleural, atrial, ureteral, fallopian, and gallbladder placement. In medically complex patients the sites for cerebrospinal fluid (CSF) diversion can be exhausted. We present a case where open retroperitoneal inferior vena cava cannulation was used for successful atrial catheter placement in a 17-month-old female. The patient had a complex abdominal, pulmonary, and vascular history precluding placement of the distal catheter in other sites or atrial placement through more peripheral venous cannulation. The patient underwent uncomplicated open retroperitoneal exposure of her inferior vena cava (IVC) with cannulation and placement of atrial catheter under fluoroscopic guidance. At the follow-up one year after surgery, the patient did not require revision with appropriate placement of the distal atrial catheter.

## Introduction

The global incidence of hydrocephalus is estimated to be 84 per 100,000 cases with the incidence of congenital hydrocephalus estimated to range from 68 to 316 cases per 100,000 [1,2]. Hydrocephalus accounts for 2.2 billion dollars of healthcare spending in the US with 1.1 billion dollars associated with readmissions alone [3]. Endoscopic third ventriculostomy (with or without choroid plexus coagulation) and ventriculoperitoneal shunting (VPS) remain the mainstay of treatment for hydrocephalus. Nearly 30% of ventriculoperitoneal shunts fail within the first year, requiring revision [4]. Reasons for VPS revision include infection, migration, over-drainage, fracture, and obstruction [5-7]. Given the high complication rates and need for revision, multiple sites for distal cerebrospinal fluid (CSF) diversion has been described when the peritoneum is no longer suitable for catheter placement including placement in the pleura, gallbladder, ureter, fallopian tube, and atrium of the heart [8-12]. Ventriculoatrial shunts (VAS) have typically been placed through cannulation of peripheral veins. When these access points have been exhausted, methods have been described that use femoral access [13]. We present a case where open retroperitoneal inferior vena cava (IVC) cannulation was used for successful atrial catheter placement.

## Case presentation

History

A 24-week premature female with a history of intraventricular haemorrhage of prematurity and bowel perforation requiring ostomy diversion underwent ventricular access device placement at 10 weeks. The VAS placement at four months was complicated by infection. After clearing her infection, she required multiple VAS revisions until attempted peritoneal placement was complicated by ostomy breakdown, which required externalization and eventual atrial reinternalization. Throughout the patient’s hospitalization, she required anticoagulation for thrombosis in multiple vessels including the left common and proximal femoral artery, left and right iliac vein, left common femoral vein, and right internal jugular vein. At 17 months, during repeat attempted distal revision of her VAS, no central venous access could be obtained and the shunt was left externalized. Magnetic resonance venography (MRV) demonstrated patent IVC. Given the patient’s complicated abdominal history, chronic lung disease, and inability to obtain central access through peripheral cannulation, the decision was made to undergo retroperitoneal exposure and direct access to her IVC.

Operation

**Figure 1:**
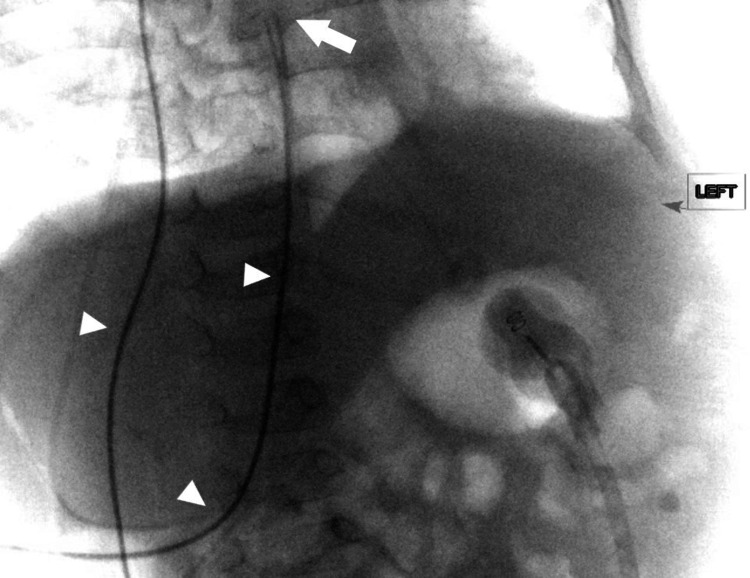
Intraoperative fluoroscopy with omnipaque in the distal catheter (arrowheads) demonstrating cannulation of IVC with termination in the right atrium (arrow). IVC: Inferior vena cava

The patient was positioned supine with a roll underneath her right shoulder and abdomen with her head turned to the left. The head, neck, abdomen, and flank were prepped and draped in a sterile fashion. A transverse flank incision was made and the external oblique divided. Dissection was carried through the abdominal wall muscles until the transversalis was exposed and divided to enter the retroperitoneal space. The peritoneum was mobilized anteriorly, and the psoas muscle was identified posterolaterally. Two vessel loops were placed around the IVC. The distal valve stem at the head was then exposed and a new distal catheter was tunnelled to the flank incision. A venotomy was made into the IVC and the distal catheter was introduced. Under fluoroscopic guidance, the catheter was threaded to the right atrium (Figure 1). Blood return was ensured and the distal catheter was then flushed. A purse-string suture was used to secure the catheter in place at the exit site of the IVC. The catheter was cut to the appropriate length and reconnected to the distal valve stem.

Postoperative course

**Figure 2:**
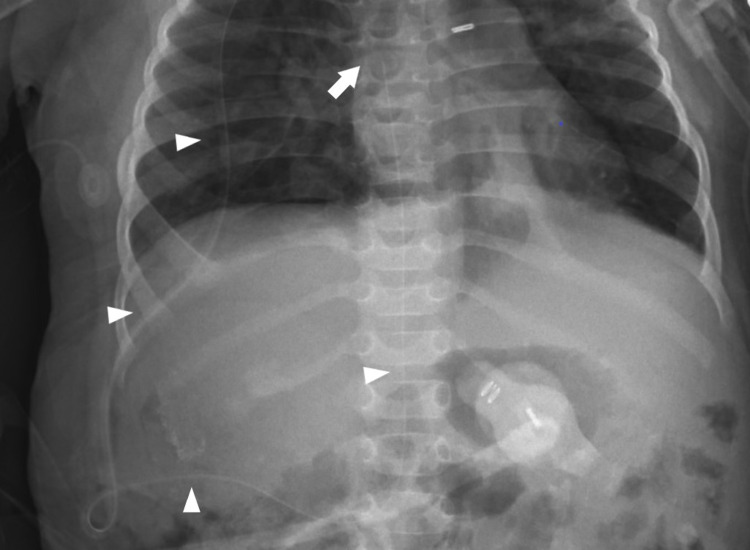
Postoperative radiograph demonstrating distal catheter coursing over the flank and entering IVC (arrowheads) with termination in the right atrium (arrow) IVC: Inferior vena cava

Postoperative imaging confirmed appropriate placement of the catheter and decompressed ventricular system (Figure 2). The patient was discharged home, with interval imaging at six months demonstrating a functioning VAS and continued appropriate placement of her distal catheter. The patient has not required a shunt revision for a year which is the longest period the patient has gone without the need for shunt revision since birth.

## Discussion

Nulsen and Spitz described the use of a unidirectional flow regulating valve to divert CSF from the ventricle to the jugular vein through open dissection [14]. Open dissection of the jugular or facial vein transitioned to the current adoption of percutaneous cannulation for atrial catheter placement after it was first described in 1981 [15]. In patients where the superior vena cava (SVC) system cannot be utilized, femoral vein insertion of the catheter has been described [13]. Given thrombi in the patient’s bilateral iliac veins and SVC system, previously described cannulation could not be used to obtain vascular access. The patient's significant abdominal and pulmonary history precluded peritoneal, gallbladder, or pleural placement while direct cardiac placement was deemed too extensive of a procedure for our patient with multiple significant comorbidities.

Ureteral shunts have only been described in patients as young as five years old, significantly older than our patient [12]. Although no longer requiring nephrectomy, the limited number of ureteric shunts described in the literature have been complicated by vesicular stones, recurrent meningitis, hypokalemia, wound infection, and erosion of the ureteral wall [10]. Due to the complication profile, higher risk of vesicular stones in our patient due to pamidronate administration, and the risk of future volume and electrolyte depletion, ureteral placement was deemed unsuitable.

Despite well-tolerated placement, removal of the distal catheter from the IVC in the event of revision carries with it a risk of retroperitoneal hematoma. While removal of the catheter without open exposure will likely lead to limited venous bleeding tamponaded by surrounding tissue and post-surgical scarring, the risk of significant haemorrhage is unknown. Removal of percutaneously placed trans-lumbar IVC catheters used for long-term central access has been shown to have a low risk of significant haemorrhage. However, these procedures do not involve exposure or dissection [16,17]. Such percutaneous cannulation of the IVC may be a feasible, less invasive approach for atrial catheter placement. Trans-lumbar venous catheters were associated with thrombosis related dysfunction in eight out of 40 patients, were responsive to thrombolytic therapy and remained in place for up to 137 days. More investigation is needed to determine if trans-lumbar IVC cannulation for atrial catheter placement is achievable.

While IVC cannulation was well-tolerated initially, at the 12-month follow-up the long-term course is unknown. With patient growth, the migration of the distal catheter will need to be followed and the need for lengthening assessed. Due to the caudal and the rostral course of the distal catheter, shortening will occur at twice the rate of growth as seen in ventriculofemoralatrial shunts [13]. Nevertheless, migration should overall be less than the ventriculofemoralatrial shunt due to the overall lower distance traversed by the catheter. 

The retroperitoneal exposure described shares similarities used by neurosurgeons and access surgeons during oblique lumbar exposure. Manipulation of the IVC carries with it risks of potential catastrophic haemorrhage. The comparatively low risk of lumbar plexopathy from an oblique lumbar exposure is likely lower in the above exposure due to the limited need for retraction of the psoas [18]. Additionally, peritoneal manipulation carries with it a risk of postoperative ileus, not experienced in our patient [19].

## Conclusions

In the present case report, we describe the surgical management of a patient with complex medical and shunt history. In cases where typical sites for CSF diversion are not suitable, assessment of possible locations for distal catheter placement may include a multidisciplinary team involving general surgeons, cardiothoracic surgeons, and even urologic surgeons. During retroperitoneal exposure, care must be taken to avoid IVC injury. Pediatric patients with the placement of an atrial catheter through cannulation of the IVC must be monitored to account for their growth and care must be taken when the need for revision arises.

## References

[1] Szuflita N, Kirkham A, Gerald T (2021). Native ureter ventriculo-ureteral shunt placement for management of refractory hydrocephalus in a child with a history of renal transplant: case report and technical note. Urology.

[2] HA GR 3rd (1954). Peritoneal shunt for hydrocephalus, utilizing the fimbria of the fallopian tube for entrance to the peritoneal cavity. J Neurosurg.

[3] Smith GW, Moretz WH, Pritchard WL (1958). Ventriculo-biliary shunt; a new treatment for hydrocephalus. Surg Forum.

[4] Weinman D, Paul AT (1967). Ventriculoauriculostomy for infantile hydrocephalus using a direct cardiac approach. Technical note. J Neurosurg.

[5] Subramaniam V, Ganapathy S, Paruchuri S (2020). Ventriculo-ureteric shunts, the last resort in complicated shunt patients. Interdisciplinary Neurosurgery.

[6] Isaacs AM, Riva-Cambrin J, Yavin D (2018). Age-specific global epidemiology of hydrocephalus: systematic review, metanalysis and global birth surveillance. PLoS One.

[7] Dewan MC, Rattani A, Mekary R (2018). Global hydrocephalus epidemiology and incidence: systematic review and meta-analysis. J Neurosurg.

[8] LeHanka A, Piatt J (2020). Readmission and reoperation for hydrocephalus: a population-based analysis across the spectrum of age. J Neurosurg.

[9] Al-Tamimi YZ, Sinha P, Chumas PD (2014). Ventriculoperitoneal shunt 30-day failure rate: a retrospective international cohort study. Neurosurgery.

[10] Furtado LMF, Da Costa Val Filho JA, Faleiro RM, Vieira JAL, Dantas Dos Santos AK (2021). Abdominal complications related to ventriculoperitoneal shunt placement: a comprehensive review of literature. Cureus.

[11] Hanak BW, Bonow RH, Harris CA, Browd SR (2017). Cerebrospinal fluid shunting complications in children. Pediatr Neurosurg.

[12] Pan P (2018). Outcome analysis of ventriculoperitoneal shunt surgery in pediatric hydrocephalus. J Pediatr Neurosci.

[13] Philips MF, Schwartz SB, Soutter AD, Sutton LN (1997). Ventriculofemoroatrial shunt: a viable alternative for the treatment of hydrocephalus. Technical note. J Neurosurg.

[14] Nulsen FE, Spitz EB (1951). Treatment of hydrocephalus by direct shunt from ventricle to jugular vain. Surg Forum.

[15] Ashker K, Fox JL (1981). Percutaneous technique for insertion of an atrial catheter for CSF shunting. Technical note. J Neurosurg.

[16] Lund GB, Lieberman RP, Haire WD, Martin VA, Kessinger A, Armitage JO (1990). Translumbar inferior vena cava catheters for long-term venous access. Radiology.

[17] Azizkhan RG, Taylor LA, Jaques PF, Mauro MA, Lacey SR (1992). Percutaneous translumbar and transhepatic inferior vena caval catheters for prolonged vascular access in children. J Pediatr Surg.

[18] Xu DS, Walker CT, Godzik J, Turner JD, Smith W, Uribe JS (2018). Minimally invasive anterior, lateral, and oblique lumbar interbody fusion: a literature review. Ann Transl Med.

[19] Park SC, Chang SY, Mok S, Kim H, Chang BS, Lee CK (2021). Risk factors for postoperative ileus after oblique lateral interbody fusion: a multivariate analysis. Spine J.

